# Analysis of the disease burden of cardiomyopathy in children aged 0–14 years in China from 1990 to 2019

**DOI:** 10.3389/fpubh.2023.1198924

**Published:** 2023-08-04

**Authors:** Qingyu Kong, Meng Li, Minmin Wang, Haizhao Zhao, Xiaorong Yang, Cuifen Zhao

**Affiliations:** ^1^Department of Pediatric Cardiology, Qilu Hospital of Shandong University, Jinan, Shandong, China; ^2^Clinical Epidemiology Unit, Qilu Hospital of Shandong University, Jinan, Shandong, China; ^3^Clinical Research Center of Shandong University, Qilu Hospital, Cheeloo College of Medicine, Shandong University, Jinan, Shandong, China

**Keywords:** cardiomyopathy, prevalence, mortality, disability-adjusted life years, children, China

## Abstract

**Objectives:**

To assess the disease burden and changing trend of cardiomyopathy in children aged 0–14 years in China from 1990 to 2019.

**Methods:**

This study was based on the Global Burden of Disease Study 2019; the age-specific prevalence rate, mortality rate and disability-adjusted life year (DALY) rate were used for analysis. Estimated annual percentage change (EAPC) in burden rate and its 95% confidence interval were calculated. The data of China were compared with the global average level.

**Results:**

In 2019, the numbers of prevalence, deaths, and DALYs of cardiomyopathy in children aged 0–14 years in China were 4,493 [95% uncertainty interval (*UI*): 2687 ~ 6,838], 434 (95%*UI*: 337 ~ 565) and 37,522 (95%*UI*: 29,321 ~ 48,891), with declining amplitudes of 16.32, 70.56, and 70.74%, compared with 1990, respectively. In 2019, the prevalence rate of cardiomyopathy in Chinese children aged 0–14 years was 2.00/100,000 (95%*UI*: 1.2/100,000 ~ 3.04/100,000), higher than 1990 [1.66/100,000 (95%*UI*:1.00/100,000 ~ 2.53/100,000)]; mortality rate was 0.19/100,000 (95%*UI*: 0.15/100,000 ~ 0.25/100,000), significantly lower than 1990 [0.46/100,000 (95%*UI*: 0.25/100,000 ~ 0.95/100,000)]; DALY rate was 16.69/100,000 (95%*U*I: 13.04/100,000 ~ 21.75/100,000), also significantly lower than 1990 [39.71/100,000 (95%*UI*: 22.06/100,000 ~ 82.8/100,000)]. All burden rates of cardiomyopathy in Chinese children aged 0–14 years old were all lower than the global averages of 2019; the burden rates of male children were higher than female children. In all calendar years from 1990 to 2019, the mortality and DALY rates of children younger than 1-year-old were significantly higher than in the other age groups of 0–14 years old. From 1990 to 2019, the prevalence rate of cardiomyopathy aged 0–14 years old gradually increased, with EAPC of 0.82 (95%*CI*: 0.71–0.93); mortality rate and DALY rate decreased [EAPC = −2.32 (95%*CI*: −2.59 to −2.05)].

**Conclusion:**

From 1990 to 2019, the disease burden of cardiomyopathy in children of China aged 0–14 years was heterogeneous; the burden of male children was higher than females; and the burden of cardiomyopathy in children younger than 1 year old needs more attention.

## Introduction

Cardiomyopathy is a kind of cardiovascular disease which can seriously affect the physical and mental health of children (1). It has the characteristics of high disability and mortality, with extremely poor prognosis and a huge disease burden (2, 3). Genetic variations and systemic diseases account for the main etiologies of pediatric cardiomyopathy which are complex and heterogeneous (4, 5). In pediatric cardiomyopathy, dilated cardiomyopathy is the most common, followed by hyper-trophic cardiomyopathy; restrictive cardiomyopathy and left ventricular myocardial insufficiency are relatively rare (6). Structural cardiac disease, infections causes, autoimmune diseases, drug reactions, metabolic disorders, hypersensitive reactions, and other exposures can also lead to cardiomyopathy (7, 8). Nearly 40% of cardiomyopathy patients developed sudden death or require heart transplantation in childhood or adolescence (8). The risk factors including pathogenesis, clinical manifestations, structural abnormalities, genetic phenotype are the leading reasons for poor prognosis in pediatric cardiomyopathy. According to population-based studies in the United States, Finland and Australia, the annual incidence of primary cardiomyopathy in children was about 1 in 100,000, but there were some differences among ages, genders and races (9–11). The incidence of cardiomyopathy was high in infancy, eight times higher than in older children (9).

The epidemiological characterization of pediatric cardiomyopathy in specific populations can help guide the formulation of public health policies, and further researches on prevention, diagnosis and treatment strategies. Published clinical studies related to cardiomyopathy in children are very rare, and no epidemiological studies have been published on cardiomyopathy in the population of children aged 0–14 years. The Global Burden of Disease (GBD) is currently the most influential study of disease burden in the world, and its database was updated in 2019 (12, 13). Based on GBD 2019, this study analyzed the disease burden and changing trend of cardiomyopathy in children aged 0–14 years in China from 1990 to 2019, and compared with global data, which aimed to provide a reference for guiding the research of pediatric cardiomyopathy and formulation of prevention strategies.

## Methods

### Data source

This study was based on the data of GBD 2019 from Global Health Data Exchange. The GBD 2019 has collected global disease burden data from primary research studies and various databases, and performed subsequent data analysis to provide systematical estimations on 369 diseases (12), which gives a unique opportunity to understand the latest state of cardiomyopathy in children of China. Following the methodology framework and analytical strategies used in the GBD 2019 study, this present study aimed to summarize the prevalence, deaths, and disability-adjusted life years (DALYs), and the corresponding secular trends of cardiomyopathy in children aged 0–14 years in China and worldwide from 1990 to 2019. DALY is one of the most important comprehensive indicators describing the burden of disease. And, it refers to the total healthy life years lost from morbidity to death, including the loss of healthy life caused by disease and disability (14). The Guidelines for Accurate and Transparent Health Estimates Reporting (GATHER) statement were obeyed to analyze the GBD database in every step (Report Checklist). The database was classified according to gender, age groups and years, and the burden of pediatric cardiomyopathy was analyzed using the standard model after modification. The International Classification of Disease (ICD) codes were adopted to identify cardiomyopathy in children aged 0–14 years old. The disease codes include 425.0–425.3, 425.7–425.8, 429.0 in ICD 9; I42.1–I42.5, I42.7–I42.8, I43–I43.9 in ICD 10. Statistical analysis code is freely available from the following website: http://ghdx.healthdata.org/gbd-2019/code.

### Analytical indicators

Age-specific prevalence rate (ASPR), age-specific mortality rate (ASMR), and age-specific DALY rate (ASDR) were used to assess the burden of pediatric cardiomyopathy. The estimated annual percentage change (EAPC) and its 95% confidence interval (CI) were calculated (15, 16). In this study, the burden of Chinese children aged 0–14 years from 1990 to 2019 was analyzed by gender, age groups, and calendar years, and was compared with the global data. Age grouping adopted the inherent age grouping mode of GBD 2019, including less than 1 year old, 1–4 years old, 5–9 years old, and 10–14 years old.

### Statistical analysis

Based on GBD 2019, the burden of cardiomyopathy of Chinese children aged 0–14 years from 1990 to 2019 was modeled by the Cause of Death Ensemble tool, DisMod-MR, and spatiotemporal Gaussian regression. Previous studies have described the primary data explanations and analytic approaches of the GBD 2019 study in detail (12, 13, 17). In this study, we conducted a descriptive analysis of the numbers of prevalence, deaths, and DALYs in China and the world in 1990 and 2019, and calculated the annual trends of age-specific rates (ASRs) from 1990 to 2019. EAPC was used to assess the annual trend in ASR to characterize the long-term change in the burden of pediatric cardiomyopathy. The upward trend is defined as the minimum value of EAPC and its 95% CI being positive; instead, a decreasing trend was recognized when EAPCs and the maximum of the 95% CI were negative; and the ASR trend is stable when neither meets (18, 19). Statistical analysis was completed using the R language version 4.2.1.

## Results

### Overview of the disease burden of cardiomyopathy in children aged 0–14 years in China, and comparison with global data

In 2019, the prevalent number of cardiomyopathy in children aged 0–14 in China was 4,493 [95% uncertain interval (UI): 2,687 ~ 6,838], down 16.32% compared with 1990 [5,369 (95%UI: 3221 ~ 8,164)] (Table 1); the number of deaths was 433 (95%UI: 337 ~ 565), down 70.56% compared with 1990 [1,474 (95%UI: 819 ~ 3,078)] (Table 2); the number of DALYs was 37,522 (95%UI: 29,321 ~ 48,891), down 70.74% compared with 1990 [128,224 (95%UI: 71,231 ~ 267,370)] (Table 3). In 2019, the ASPR of cardiomyopathy aged 0–14 years old was 2.00/100,000 (95%UI:1.20/100,000 ~ 3.04/100,000) in China, which was higher than in 1990 [1.66/100,000 (95%UI: 1.00/100,000 ~ 2.53/100,000)] (Table 1). In contrast, the global ASPR [3.21/100,000 (95%UI:1.69/100,000 ~ 5.21/100,000)] decreased compared with 1990 [3.55/100,000 (95%UI: 2.21/100,000 ~ 5.30/100,000)] (Table 1). ASMR and ASDR of 2019 in China were 0.19/100,000 (95%UI: 0.15/100,000 ~ 0.25/100,000) and 16.69/100,000 (95%UI: 13.04/100,000 ~ 21.75/100,000), both decreasing compared with the corresponding data of 0.46/100,000 (95%UI: 0.25/100,000 ~ 0.95/100,000) and 39.71/100,000 (95%UI: 22.06/100,000 ~ 82.80/100,000) in 1990 (Tables 2, 3). Both the ASMR and the ASDR in 2019 also declined globally compared with 1990. In 2019, the ASRs of cardiomyopathy in Chinese children aged 0–14 years old were all lower than the corresponding global averages (Tables 1–3).

**Table 1:** Prevalence and age-specific prevalence rate per 100,000 people of cardiomyopathy in children aged 0–14 years old in 1990 and 2019, and its estimated annual percentage change from 1990 to 2019.

Characteristics	1990		2019		EAPC of ASPR (95%CI) from 1990 to 2019
	Prevalence (95%UI)	ASPR/100,000 (95%UI)	Prevalence (95%UI)	ASPR/100,000 (95%UI)	
China					
Both	5,369 (3,221~8,164)	1.66 (1.00~2.53)	4,493 (2,687~6,838)	2.00 (1.2~3.04)	0.82 (0.71~0.93)
Male	3,167 (1,889~4,818)	1.88 (1.12~2.86)	2,711 (1,613~4,109)	2.23 (1.33~3.38)	0.74 (0.64~0.83)
Female	2,201 (1,331~3,344)	1.43 (0.86~2.16)	1,782 (1,080~2,732)	1.72 (1.04~2.64)	0.90 (0.78~1.02)
Age groups					
<1	178 (97~301)	0.76 (0.41~1.28)	134 (73~227)	0.89 (0.49~1.51)	0.73 (0.62~0.84)
1~4	1,910 (955~3,140)	1.86 (0.93~3.05)	1,535 (772~520)	2.17 (1.09~3.57)	0.92 (0.8~1.04)
5~9	1,659 (857~2,747)	1.58 (0.82~1.62)	1,401 (717~2,295)	1.93 (0.99~3.16)	0.93 (0.81~1.05)
10~14	1,621 (931~585)	1.76 (1.01~2.81)	1,424 (820~2,256)	2.14 (1.23~3.4)	0.69 (0.59~0.79)
Global					
Both	62,240 (38,833~92,899)	3.55 (2.21~5.30)	20,606 (10,825~33,441)	3.21 (1.69~5.21)	−0.23 (−0.26 to −0.20)
Male	35,346 (22,187~52,469)	3.92 (2.46~5.83)	11,880 (6,170~19,101)	3.59 (1.86~5.76)	−0.23 (−0.25 to −0.20)
Female	26,895 (16,569~40,421)	3.15 (1.94~4.74)	8,726 (4,583~14,302)	2.81 (1.47~4.60)	−0.23 (−0.27 to −0.19)
Age groups					
<1	1,818 (1,003~3,042)	1.38 (0.76~2.31)	2,064 (1,123~3,497)	1.56 (0.85~2.65)	0.42 (0.40~0.45)
1~4	18,812 (10,853~29,856)	3.76 (2.17~5.97)	20,863 (11,788~33,511)	3.93 (2.22~6.31)	0.15 (0.13~0.16)
5~9	20,823 (11,438~34,459)	3.56 (1.95~5.89)	22,043 (11,987~36,886)	3.37 (1.83~5.63)	−0.24 (−0.28 to −0.2)
10~14	20,787 (11,061~33,358)	3.87 (2.06~6.22)	20,606 (10,825~33,441)	3.21 (1.69~5.21)	−0.65 (−0.71 to −0.59)

ASPR, age-specific prevalence rate; UI, uncertainty interval; EAPC, estimated annual percentage change; CI, confidential interval.

**Table 2:** Deaths and age-specific mortality rate per 100,000 people of cardiomyopathy in children aged 0–14 years old in 1990 and 2019, and its estimated annual percentage change from 1990 to 2019.

Characteristics	1990		2019		EAPC of ASMR (95%CI) from 1990 to 2019
	Deaths (95%UI)	ASMR/100,000 (95%UI)	Deaths (95%UI)	ASMR/100,000 (95%UI)	
China					
Both	1,474 (819~3,078)	0.46 (0.25~0.95)	433 (337~565)	0.21 (0.15~0.31)	−2.32 (−2.59 to −2.05)
Male	773 (446~1,991)	0.46 (0.26~1.18)	260 (185~377)	0.17 (0.12~0.22)	−1.98 (−2.28 to −1.68)
Female	701 (329~1,687)	0.45 (0.21~1.09)	173 (127~226)	0.19 (0.15~0.25)	−2.78 (−3.02 to −2.54)
Age groups					
<1	965 (511~2,155)	4.11 (2.18~9.18)	245 (188~330)	1.62 (1.25~2.19)	−2.88 (−3.36 to −2.40)
1~4	106 (66~196)	0.33 (0.17~0.69)	59 (45~78)	0.12 (0.09~0.17)	−3.39 (−3.79 to −2.99)
5~9	102 (62~198)	0.10 (0.06~0.19)	49 (36~65)	0.07 (0.05~0.09)	−1.39 (−1.87 to −0.91)
10~14	300 (156~630)	0.10 (0.06~0.19)	81 (61~110)	0.08 (0.06~0.11)	−0.60 (−1.05 to −0.16)
Global					
Both	10,986 (7,979~16,654)	0.63 (0.46~0.95)	7,163 (5,511~9,300)	0.37 (0.28~0.47)	−1.55 (−1.68 to −1.41)
Male	5,707 (3,586~10,025)	0.63 (0.40~1.11)	3,896 (2,874~5,342)	0.39 (0.28~0.53)	−1.38 (−1.52 to −1.24)
Female	5,279 (3,828~8,453)	0.62 (0.45~0.99)	3,268 (2,455~4,137)	0.34 (0.26~0.44)	−1.74 (−1.87 to −1.61)
Age groups					
<1	6,699 (4,897~10,120)	5.09 (3.72~7.69)	3,923 (2,986~5,166)	2.97 (2.26~3.91)	−1.67 (−1.83 to −1.51)
1~4	2,500 (1,617~4,297)	0.50 (0.32~0.86)	1,600 (1,159~2,305)	0.30 (0.22~0.43)	−1.44 (−1.55 to −1.33)
5~9	884 (653~1,395)	0.15 (0.11~0.24)	792 (635~1,001)	0.13 (0.11~0.16)	−0.74 (−0.82 to −0.65)
10~14	902 (727~1,262)	0.17 (0.14~0.24)	848 (690~1,022)	0.13 (0.11~0.16)	−0.85 (−0.92 to −0.77)

ASMR, age-specific mortality rate; UI, uncertainty interval; EAPC, estimated annual percentage change; CI, confidential interval.

**Table 3:** DALYs and age-specific DALY rate per 100,000 people of cardiomyopathy in children aged 0–14 years old in 1990 and 2019, and its estimated annual percentage change from 1990 to 2019.

Characteristics	1990	2019			EAPC of ASIR (95%CI) from 1990 to 2019
	DALYs (95%UI)	ASDR/100,000 (95%UI)	DALYs (95%UI)	ASDR/100,000 (95%UI)	
China					
Both	12,8224 (71,231~267,370)	39.71 (22.06~82.8)	37,522 (29,321~48,891)	16.69 (13.04~21.75)	−2.32 (−2.59 to −2.05)
Male	67,200 (38,789~173,243)	39.89 (23.03~102.84)	22,490 (16,058~32,629)	18.52 (13.22~26.86)	−1.99 (−2.29 to −1.69)
Female	61,024 (28,609~147,143)	39.51 (18.52~95.26)	15,033 (11,048~19,608)	14.55 (10.69~18.98)	−2.77 (−3.01 to −2.54)
Age groups					
<1	85,413 (45,262~190,660)	363.89 (192.83~812.28)	21,660 (16,660~29,212)	143.75 (110.57~193.87)	−2.88 (−3.36 to −2.40)
1~4	8,297 (5,235~15,134)	28.28 (14.73~59.11)	4,660 (3,585~6,073)	10.65 (8.12~14.42)	−3.35 (−3.74 to −2.96)
5~9	8,524 (5,214~16,291)	8.14 (4.98~15.55)	4,128 (3,084~5,448)	5.68 (4.25~7.50)	−1.34 (−1.81 to −0.87)
10~14	25,991 (13,538~54,332)	8.07 (5.09~14.73)	7,075 (5,396~9,580)	6.60 (5.07~8.59)	−0.58 (−1.01 to −0.14)
Global					
Both	955,140 (693,517~1,441,833)	54.46 (39.54~82.20)	620,609 (479,373~804,032)	31.69 (24.46~41.03)	−1.55 (−1.69 to −1.42)
Male	496,340 (311,203~869,370)	55.11 (34.55~96.52)	337,397 (250,069~466,822)	33.35 (24.72~46.14)	−1.39 (−1.53 to −1.25)
Female	458,800 (333,448~734,021)	53.77 (39.08~86.02)	283,212 (212,319~358,544)	29.87 (22.40~37.82)	−1.74 (−1.87 to −1.61)
Age groups					
<1	593,190 (433,813~895,811)	450.82 (329.69~680.81)	347,514 (264,669~457,588)	263.3 0 (200.53~346.70)	−1.67 (−1.83 to −1.51)
1~4	216,833 (140,711~371,612)	43.32 (28.11~74.24)	139,589 (101,670~200,047)	26.30 (19.15~37.68)	−1.43 (−1.53 to −1.32)
5~9	74,226 (55,324~116,152)	12.68 (9.45~19.85)	66,818 (53,355~83,864)	10.21 (8.15~12.81)	−0.72 (−0.81 to −0.64)
10~14	70,891 (57,825~98,817)	13.71 (10.95~16.81)	66,689 (55,011~79,796)	10.38 (8.57~ 12.43)	−0.84 (−0.92 to −0.76)

DALY, disability-adjusted life year; ASDR, age-specific DALY rate; UI, uncertainty interval; EAPC, estimated annual percentage change; CI, confidential interval.

### Gender characteristics of the disease burden of cardiomyopathy in children aged 0–14 years in China, and comparison with global data

**Figure 1:**
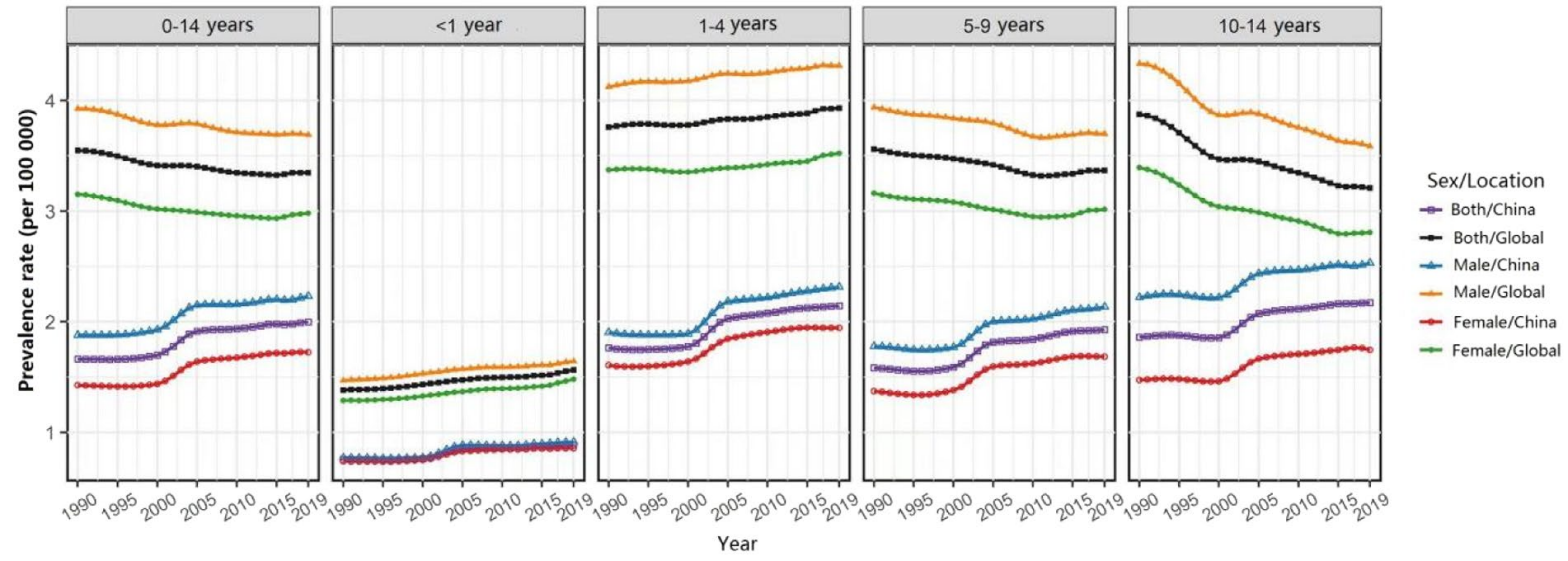
Changes in the age-specific prevalence rate of children with cardiomyopathy aged 0–14 years across all age groups and genders, China and the world, from 1990 to 2019.

**Figure 2:**
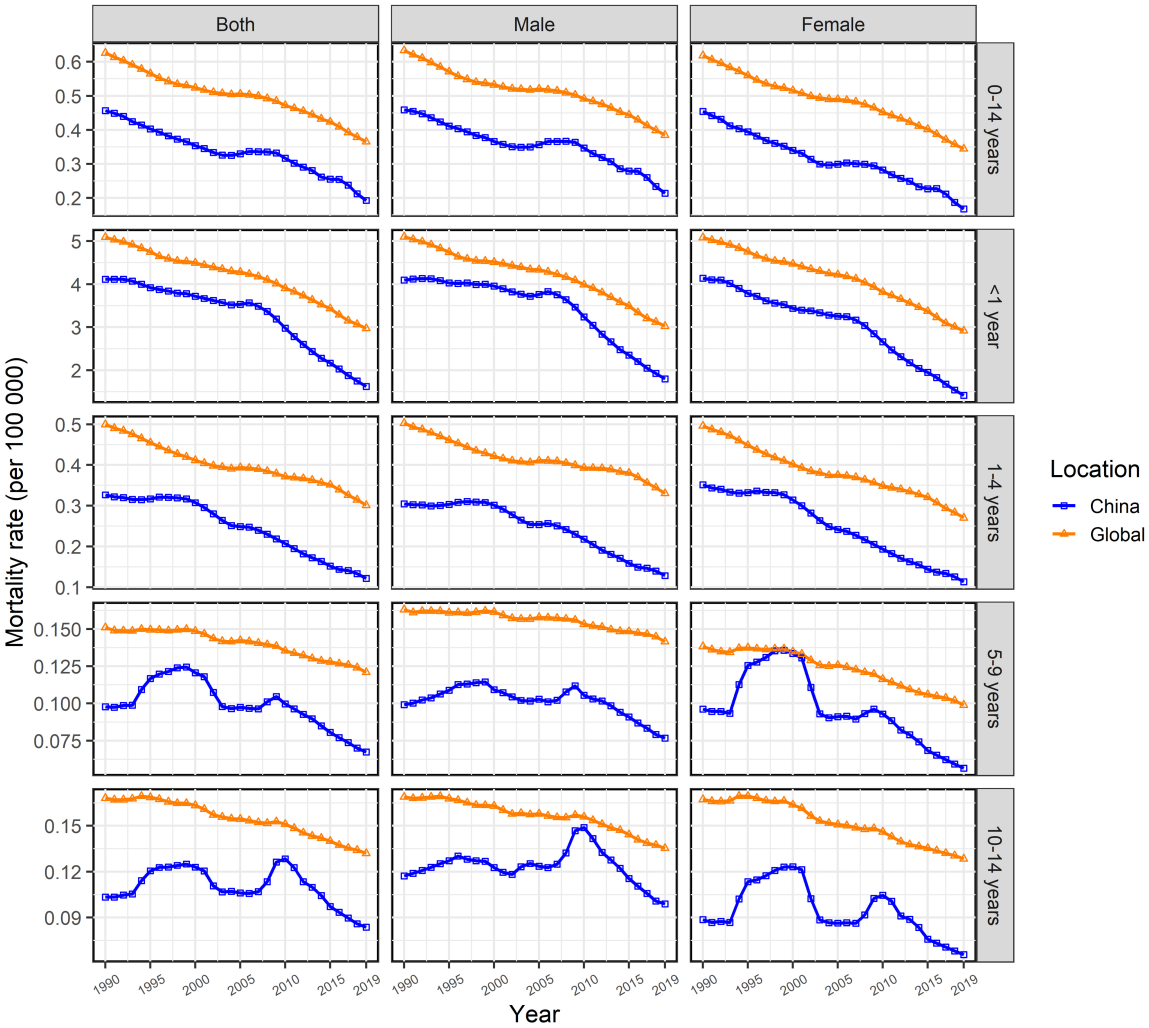
Changes in the age-specific mortality rate of children with cardiomyopathy aged 0–14 years across all age groups and genders, China and the world, from 1990 to 2019.

**Figure 3:**
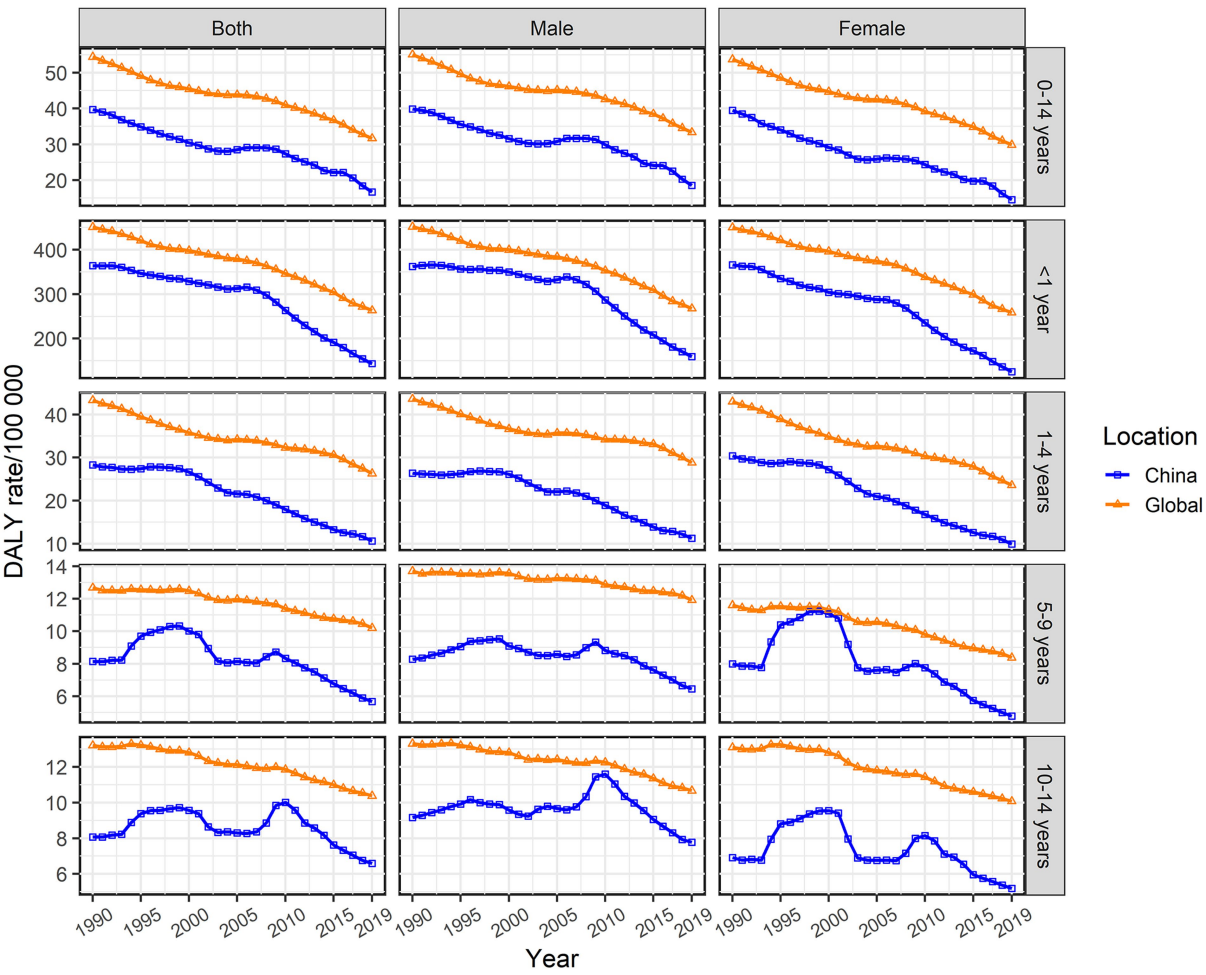
Changes in the age-specific disability-adjusted life year (DALY) rate of children with cardiomyopathy aged 0–14 years across all age groups and genders, China and the world, from 1990 to 2019.

In 2019, the prevalence of Chinese male children aged 0–14 with cardiomyopathy was 2,711 (95%UI: 1,613–4,109), and 1782 (95%UI: 1,080–2,732) in women; the number of deaths in Chinese male children with cardiomyopathy was 260 (95%UI:185 ~ 377), and DALYs were 22,490 (95%UI: 16,058 ~ 32,629); the number of deaths in Chinese female children with cardiomyopathy was 173 (95%UI: 127 ~ 226), and DALYs were 15,033 (95%UI: 11,048 ~ 19,608) (Tables 1–3). In 2019, the ASPR, ASMR, and ASDR of cardiomyopathy in male children aged 0–14 years in China were 2.23/100,000 (95%UI: 1.33/100,000 to 3.38/100,000), 0.21/100,000 (95%UI: 0.15/100,000 to 0.31/100,000), and 18.52/100,000 (95%UI: 3.22/100,000 to 26.86/100,000), which were higher than the corresponding values of female children [1.72/100,000 (95%UI: 1.04/100,000 to 2.64/100,000), 0.17/100,000 (95%UI: 0.12/100,000 to 0.22/100,000), and 14.55/100,000 (95%UI: 10.69/100,000 to 18.98)/100,000] (Tables 1–3). In all calendar years from 1990 to 2019, the ASPR, ASMR and ASDR of Chinese children with cardiomyopathy aged 0–14 years old in males were higher than that in females; and the ASRs of cardiomyopathy in Chinese children aged 0–14 years old were all lower than the global averages of the corresponding genders (Figures 1–3).

### Characteristics of the disease burden of cardiomyopathy in children aged 0–14 years in China in age groups, and comparison with global data

In 2019, the ASPR of cardiomyopathy of all age groups among 0–14 years old in China increased compared with 1990, while ASMR and ASDR decreased compared with 1990. The highest ASPR in 1990 and 2019 were found in the 1–4 years old group (Table 1), and the highest ASMR and ASDR were found in the <1 year age group (Tables 2, 3). It is noteworthy that the number of deaths in children in the age group less than 1 year exceeded the number of prevalence in 1990 and 2019, more obviously in 1990; while the opposite results were observed in the other age groups (Table 2). From 1990 to 2019, ASPR, ASMR and ASDR of Chinese children with cardiomyopathy in all age groups among 0–14 years old were lower than the global average of the corresponding age groups (Figures 1–3).

### Annual trends in the disease burden of cardiomyopathy in children aged 0–14 years old in China, and comparison with global data

From 1990 to 2019, the ASPR of cardiomyopathy in children aged 0–14 years in China gradually increased with an EAPC of 0.82 (95% CI: 0.71–0.93); the upward trend of male children [EAPC = 0.74 (95% CI: 0.64–0.83)] is weaker than that of female children [EAPC = 0.9(95% CI: 0.78–1.02)] (Table 1). However, the ASPR of cardiomyopathy in children aged 0–14 years old has shown a downward trend globally with similar trends for both male and female children (Table 1). From 1990 to 2019, the ASMR and ASDR of cardiomyopathy in children aged 0–14 years old in China showed a downward trend with the same EAPC of [−2.32 (95% CI: −2.59 to −2.05)], which was oblivious than the global trend [EAPC = −1.55 (95% CI: −1.68 to −1.41)] (Tables 2, 3). In Chinese male children aged 0–14 years old, ASMR and ASDR showed a downward trend [EAPC = −1.98 (95% CI: −2.28 ~ −1.68) and − 1.99 (95% CI: −2.29 ~ −1.69)], with a weaker degree than in female children [EAPC = −2.78 (95% CI: −3.02 ~ −2.54) and − 2.77 (95% CI: −3.01 ~ −2.54)] (Tables 2, 3). During the period 1990–2019, the two age groups with the most obvious upward trend of ASPR were 5–9 years old and 1–4 years old; 1–4 years old and younger than 1 year old are the two age groups with the most significant downward trend in ASMR and ASDR. The changing trend of ASMR was highly consistent with ASDR in all age groups between 0 and 14 years old in China and the whole world (Figures 2, 3).

## Discussion

This study found that the prevalence of children with cardiomyopathy aged 0–14 years in China decreased from 1990 to 2019, but ASPR showed an upward trend; the number of deaths, DALYs, ASMR, and ASDR all showed a downward trend. The results suggested that the overall burden of cardiomyopathy in children aged 0–14 years in China presented a downward trend during the period of 1990–2019, which was mainly due to the continuous progress of basic, translational, and clinical researches related to cardiomyopathy. Genomics technology has promoted the continuous development of precision medicine, and more and more unexplained cardiomyopathy has been unveiled (20–24). In this context, medical personnel and scientific researches have gained a deeper understanding on the causes of pediatric cardiomyopathy, and the diagnosis and treatment strategies for pediatric cardiomyopathy have also been continuously developed and improved. Meanwhile, we should also pay attention to the rising trend of ASPR in children aged 0–14 years with cardiomyopathy in China, which was mainly due to the significant decline in ASMR. The continuous progress in diagnosis and treatment significantly reduced the mortality rate of children with cardiomyopathy. The continuous improvement in precision medicine for cardiomyopathy has enabled more children with cardiomyopathy to receive timely and effective diagnosis and treatment (25). The change in the overall size of the 0–14 year old population was another important influencing factor for the change in ASPR in China. This study also found that from 1990 to 2019, children with cardiomyopathy of all age groups and different genders in China showed the same changes as the overall trend. Namely, ASPR showed an upward trend, while ASMR and ASDR showed a highly consistent downward trend. Moreover, the overall disease burden of cardiomyopathy in Chinese children aged 0–14 years was lower than the global average. China has achieved good results in the prevention and treatment of pediatric cardiomyopathy for reducing the burden, which was mainly due to the in-depth research, experience sharing, and hard work of domestic medical and scientific researchers in the diagnosis and treatment of pediatric cardiomyopathy. The birth rate inevitably affects the number of children with cardiomyopathy, therefor different birth rate in the Chinese population compared to the global one is also an important factor affecting the discrepancies in cardiomyopathy burden. Another, the incidence rate of cardiomyopathy may vary among different ethnic groups.

Gender is an important factor affecting the burden of pediatric cardiomyopathy (26). The results of this study suggested that the burden of cardiomyopathy in Chinese male children aged 0–14 years was higher than that in female children. The number of prevalence, deaths, DALYs, and their corresponding specific rates in male children were higher than those in female children. Many children with cardiomyopathy have a familial basis, and most of the related genes are autosomal with dominant transmission mainly (27). Considering the impact of genetic patterns, the burden of disease should be similar for men and women, but this contradicts clinical statistics. The gender difference may be related to the penetrance of cardiomyopathy-related genes, mitochondrial genetics, modified genes, epigenetics, environmental factors, etc. between different sexes. Functional analysis of gene expression patterns showed that female-specific deregulation genes were mainly involved in energy metabolism, transcription, and translation regulation, while male-specific deregulation genes were related to myocardial contraction (28). Sex hormones can directly affect cardiac function by influencing endothelial cell function and vascular tension through androgen and estrogen receptors present in vascular endothelium, smooth muscle cells, and cardiac fibroblasts. Previous studies had found that heart failure with preserved ejection fraction was more common in women, and women experienced heart failure later than men (29). Sex hormones also have significant impacts on the immune system. Estrogen is more inclined to produce a protective immune response to the myocardium, while androgen can increase the Th1 response and promote inflammation (30). In terms of myocardial remodeling, animal studies had shown that testosterone was the cause of poor myocardial remodeling in men; elevated testosterone levels could promote myocarditis and fibrosis, leading to increased myocardial disease and heart failure (31). Currently, there is no definite evidence that standard heart failure therapies differ significantly between males and females. Overall, the prognosis of women with cardiomyopathy is better than men, but the mechanisms are complex and multifaceted. Research on the gender-specific mechanisms of cardiomyopathy can help improve diagnostic strategies and clinical management, and in-depth research on the cellular mechanisms of the “gender effect” can help find better-targeted therapies for cardiomyopathy.

The burden of childhood cardiomyopathy has significant age characteristics. This study found that the highest ASPR for cardiomyopathy in children aged 0–14 years in China in 1990 and 2019 presented in the 1–4 year old age group, while the highest ASMR and ASDR showed in the younger than 1 year old age group. It was also found that the number of deaths among children younger than 1 year old age group exceeded the prevalence in 1990 and 2019, especially in 1990; while the number of deaths among other age groups was smaller than the prevalence in both years. Cardiomyopathy is one of the most important causes of cardiac insufficiency and sudden cardiac death in children. Many children with cardiomyopathy often have severe clinical manifestations such as heart failure, malignant arrhythmia, embolism, or cardiogenic shock at the time of diagnosis, with extremely poor prognosis and high mortality. The result that the number of deaths in children younger than 1 year old was higher than the prevalence further confirmed the poor prognosis of infantile cardiomyopathy. The highest mortality and DALY rates in infants were mainly related to triggers of infant cardiomyopathy, including genetic abnormalities, genetic and metabolic diseases, and congenital factors such as intrauterine infections. In addition, there is fewer safe and effective interventions for cardiac insufficiency that has been identified during the fetal period which is an important influencing factor for the high mortality rate of children after birth. The highest prevalence rate among children aged 1–4 years was mainly due to the stable survival of infants with cardiomyopathy after timely diagnosis and effective treatment. Also, the higher diagnostic rate among children aged 1–4 years with cardiomyopathy was another important reason. According to the survey and analysis of 4,981 hospitalized children’s cardiomyopathy in 33 hospitals in China from 2006 to 2018 by the Children’s Cardiology Precision Diagnosis and Treatment Cooperation Group of Pediatric Society of Chinese Medical Association, 65.15% of children with cardiomyopathy were under the age of 3 years old (32). The result suggested that the incidence rate of early childhood cardiomyopathy in China was significantly higher than that of older children, similar to foreign literature, and also similar to the statistical results of our study. In order to reduce the burden of childhood cardiomyopathy, it is necessary to pay attention to the prevention and treatment of infant cardiomyopathy, and continuously improve the status of genetic testing in the diagnosis and classification of cardiomyopathy. Emphasis should be placed on genetic counseling and eugenics, especially for high-risk families. Complete family lineage excavation and comprehensive genetic counseling should be conducted in these families.

Clinical researches and epidemiological investigations related to cardiomyopathy in children are rare, and relevant academic conferences are rarely held. In this context, our study has some strengths. First, based on the GBD 2019, the data used in our study is calculated using a robust method, and its quality is currently the best (16, 33). Second, this study firstly conducted a comprehensive analysis of the disease burden of cardiomyopathy in children aged 0–14 years in China, which is the first nationwide epidemiological survey of cardiomyopathy in children aged 0–14 years. The purpose of this study is to provide evidence-based medical evidence for future research priorities in children with cardiomyopathy in China, in order to achieve early diagnosis, ameliorate clinical outcomes, and improve the quality of life of children and their families. The epidemiological investigation of childhood cardiomyopathy has also received increasing attention in China. The Collaborative Group on Accurate Diagnosis and Treatment of Childhood Cardiomyopathy of the Cardiovascular Science Group of the Pediatrics Branch of the Chinese Medical Association conducted multicenter retrospective analyses of the clinical data of children with cardiomyopathy from 2006 to 2016 and 2008–2018, providing a reference for accurate diagnosis and treatment of children with cardiomyopathy (32, 34). Important diagnostic and therapeutic recommendations such as “Recommendations for Genetic Testing of Cardiomyopathy in Children” and “Recommendations for the Diagnosis and Treatment of Heart Failure in Children (Revised in 2020)” have also been published (35, 36). In the foreseeable future, with the continuous improvement of gene editing technology, there will also be more and better clinical plans to treat hereditary cardiomyopathy (37). We have confidence that the disease burden caused by children with cardiomyopathy in China and the world will inevitably decrease with the efforts of medical and scientific researchers.

Some limitations should be noticed. First of all, this study uses the data from GBD 2019, which has methodological limitations of GBD 2019 itself. On the other hand, the database only includes data on overall cardiomyopathy in China, which cannot clarify the epidemiological differences between different provinces and regions. Thirdly, there is no survey data for different types of cardiomyopathy in the study. Specific epidemiological investigations targeting different etiologies and types of cardiomyopathy can help to analyze the disease burden of childhood cardiomyopathy in greater depth, but the GBD 2019 does not provide a detailed classification.

In summary, from 1990 to 2019, the disease burden of cardiomyopathy in children aged 0–14 years in China showed heterogeneous changes. The burden of cardiomyopathy in male children was higher than that in female children. Moreover, the burden of cardiomyopathy among people younger than 1-year-old in China requires more attention. Policymakers, researchers, and medical personnel should develop more effective and preventive measures in terms of primary prevention, early diagnosis and treatment, disease management, improvement of public health systems, education and awareness to reduce the burden of childhood cardiomyopathy, pay attention to changes in the epidemic characteristics, and rationally allocate limited medical resources.

## Data availability statement

The original contributions presented in the study are included in the article/Supplementary material, further inquiries can be directed to the corresponding authors.

## Ethics statement

The studies involving human participants were reviewed and approved by the Institutional Review Boards of Qilu Hospital of Shandong University with approval number KYLL-202011(KS)-239. Written informed consent from the participants’ legal guardian/next of kin was not required to participate in this study in accordance with the national legislation and the institutional requirements.

## Author contributions

QK, XY, and CZ contributed to the conception, design of the work, and results visualization. QK, ML, MW, HZ, XY, and CZ contributed to the acquisition and analysis of data for the work. QK and XY drafted the manuscript. All authors critically revised the manuscript, gave final approval, contributed to the interpretation of the results, and agreed to be accountable for all aspects of the work ensuring integrity and accuracy.

## Funding

This work was supported by the Key Research and Development Program of Shandong Province (2019GSF108186). The funders were not involved in the collection, analysis, interpretation of data, the writing, and submitting of this report.

## Conflict of interest

The authors declare that the research was conducted in the absence of any commercial or financial relationships that could be construed as a potential conflict of interest.

## Publisher’s note

All claims expressed in this article are solely those of the authors and do not necessarily represent those of their affiliated organizations, or those of the publisher, the editors and the reviewers. Any product that may be evaluated in this article, or claim that may be made by its manufacturer, is not guaranteed or endorsed by the publisher.

## Abbreviations

ASDR: Age-specific DALY rate
ASMR: Age-specific mortality rate
ASPR: Age-specific prevalence rate
ASR: Age-specific rate
CI: Confidence interval
DALY: Disability-adjusted life year
EAPC: Estimated annual percentage change
GATHER: Guidelines for Accurate and Transparent Health Estimates Reporting
GBD: Global Burden of Disease
ICD: International Classification of Disease
UI: Uncertainty interval
