# Supplementary material for: Analysis of the disease burden of cardiomyopathy in children aged 0–14 years in China from 1990 to 2019
Source: Front Public Health. 2023 Aug 4;11:1198924. doi: 10.3389/fpubh.2023.1198924 (PMC10436593; doi:10.3389/fpubh.2023.1198924)
Supplement: Supplementary file 1 [file Table_1.DOCX]

setwd("J:/ODATA/GBD/GBD-Cardiomyopathy China")

library(rJava)

library(xlsxjars)

library(xlsx)

library(ggplot2)

library(gtable)

library(grid)

library(lubridate)

library(ggplot2)

library(scales)

library(magrittr)

library(tidyr)

library(RColorBrewer)

library(dplyr)

# Read the raw data

d1<-read.csv("Cardiomyopathy China.csv", header=T,stringsAsFactors=FALSE)

d1<-subset(d1,val!=0)

str(d1)

names(d1)

names(d1)<-tolower(names(d1))

head(d1)

summary(d1)

table(d1$measure_name)

table(d1$location_name)

table(d1$sex_name)

table(d1$age_name)

table(d1$cause_name)

table(d1$metric_name)

table(d1$year,d1$measure_name)

table(d1$year,d1$age_name)

d1$sex_name2<-factor(d1$sex_name, order=T, levels=c("Both", "Male", "Female"))

d1$measure_name2<-factor(d1$measure_name,order=T, levels=c("Prevalence", "Deaths",

"YLLs (Years of Life Lost)",

"YLDs (Years Lived with Disability)",

"DALYs (Disability-Adjusted Life Years)"))

d1$metric_name<-factor(d1$metric_name,order=T)

table(d1$age_name)

d1<-d1[order(d1$year,d1$location_name, d1$age_name,d1$sex_name2, d1$measure_name, d1$metric_name),]

# EAPC

d2<-subset(d1,metric_name=="Rate")

table(d2$sex_name2)

d2$g<-paste(d2$location_name,d2$sex_name,d2$measure_name,d2$age_name)

d2$g2<-factor(d2$g,order=T)

d2$g3<-as.numeric(d2$g2)

table(d2$g3)

eapcageb<-matrix(nrow=300,ncol=8)

eapcageb

for (i in 1:300) {

db<-d2[d2$g3==i, ]

names(db)<-tolower(names(db))

db$lnvalue<-log(db$val)

fit<-lm(lnvalue~year,data=db)

eapcageb[i,1]=round((exp(coefficients(fit)[2])-1)*100,2)

eapcageb[i,2]=round((exp(confint(fit)[2, 1])-1)*100,2)

eapcageb[i,3]=round((exp(confint(fit)[2, 2])-1)*100,2)

eapcageb[i,4]=db[1,2]

eapcageb[i,5]=db[1,4]

eapcageb[i,6]=db[1,6]

eapcageb[i,7]=db[1,8]

eapcageb[i,8]=db[1,21]

}

eapcageb

colnames(eapcageb)<-c("eapc","eapcl","eapcu","measure","loc","sex","age","group")

eapcageb<-as.data.frame(eapcageb)

eapcageb$ci<-paste(as.character(eapcageb$eapc),as.character(eapcageb$eapcl),sep=" (")

eapcageb$ci<-paste(eapcageb$ci,as.character(eapcageb$eapcu),sep=", ")

eapcageb$ci<-paste(eapcageb$ci,")",sep="")

View(eapcageb)

write.csv(eapcageb,"Cardiomyopathy China-eapc.csv")
